# Medical Degrees at Cambridge

**Published:** 1910-05-14

**Authors:** 


### MEDICAL DEGREES AT CAMBRIDGE.

For nearly two years a committee of the Cambridge University Special Board for Medicine has been considering the regulations for the degrees of Bachelor of Medicine and Doctor of Medicine. In the changes made in 1900 pathology and pharmacology were included in the first part, leaving surgery, midwifery, and medicine for the second part of the final examination. This was so far satisfactory that the student gained a scientific foundation for a study of disease and of treatment; but the second part now became somewhat unwieldy. Moreover, it was thought that there was a tendency on the part of those who had passed the first part of the examination to consider that it was no longer necessary to study pathology or pharmacology, even in connection with the cases they were studying in hospital, and the result has been that in the later years too little time has been devoted to clinical and practical work and too much to the study of text-books. Under the new regulations pharmacology and general pathology, along with bacteriology, are separated from the final examination and are introduced as a second part of the second examination. This examination will be more of the nature of a test of the results of teaching, and may be taken six months after the first part—anatomy and physiology—has been passed. The final examination will be divided up as formerly, up to 1900, surgery and midwifery being included in the first part, which may be entered for, should the student wish, at the end of five years' study and two years' hospital practice. The principles and practice of physic (including the diseases of children, mental diseases, and medical jurisprudence), pathology (including hygiene and preventive medicine), and pharmacology (including therapeutics and toxicology) may be taken as the second part of the third examination after the student has passed both parts of the second M.B. examination and after he has completed three years of hospital practice. At this date, of course, should the student wish it, he may take both first and second parts. In this way the examination will not press so heavily upon men of average ability, and the arrangement will encourage them to devote themselves to a more thorough preparation for the various parts of the examination. Further, owing to the rearrangement of the times for examination in pathology and pharmacology, the student will be able to clear this examination out of the way in time for him to begin his studies in the London or other hospitals, and there will be no unnecessary loss of time during the curriculum.

In view of the increasing amount and complexity of the work involved in the examination of theses for the degree of M.D. at Cambridge, and in some measure to lighten the labours of the Regius Professor of Physic, a Degree Committee, consisting of four additional members of the Special Board for Medicine, will in future assist the Regius Professor and his assessor in dealing with theses and keeping of acts for the M.D. degree.

